# Pericarditis as a cardiac manifestation of acute leptospirosis

**DOI:** 10.1007/s15010-020-01496-3

**Published:** 2020-08-10

**Authors:** M. Zechel, M. Franz, M. Baier, S. Hagel, B. T. Schleenvoigt

**Affiliations:** 1 Institute for Infection Diseases and Infection Control, University Hospital Jena, Am Klinikum 1, 07747 Jena, Germany; 2 Clinic of Internal Medicine I, University Hospital Jena, Am Klinikum 1, 07747 Jena, Germany; 3 Institute for Medical Microbiology, University Hospital Jena, Am Klinikum 1, 07747 Jena, Germany

**Keywords:** Leptospirosis, Zoonosis, Infectious disease, Pericarditis, Triathletes

## Abstract

Leptospirosis is an infectious disease with an increasing incidence worldwide. The clinical presentation is unspecific and ranges from an asymptomatic clinical course to an acute fulminant disease. The current case report describes a 32-year-old male patient who presented with ST segment elevation in the electrocardiogram about 14 days after cross-country running. Pericarditis was diagnosed and linked to an acute leptospirosis that was serologically confirmed.

## Introduction

Leptospirosis is a zoonosis transmitted across the world. In tropical and subtropical countries, leptospirosis is particularly prevalent with an incidence of 10–100/100,000 inhabitants. In contrast, in temperate regions, the incidence is about 0.1/100,000 inhabitants [1, 2]. The infective pathogen *Leptospira interrogans* belongs to the family of Leptospiraceae. These are obligatory aerobic, hanger-like bacteria [3]. *Leptospira* sp. are found in wildlife and farm animals such as rodents, dogs, cattle and pigs. The carrier animals excrete bacteria even if they are not clinically ill. The transmission to humans occurs directly or indirectly by contaminated water. Through this, bacteria enter the organism via skin injuries or mucous membranes [4, 5]. The incubation period usually lasts 7–14 days. The clinical course ranges from mild (90%) to acute fulminant forms [3, 4]. Consequently, it is suggested that the number of unreported cases is significantly higher than the rate mentioned above [5, 6]. Generally, leptospirosis can affect every organ and there are two phases of the disease: the acute phase and the immune phase (after 1 week). Typical symptoms are flu-like illness with fever up to 40 °C, headache and myalgia. Furthermore, an exanthema as well as meningitis and meningo-encephalitis may occur. Pulmonary haemorrhage is rare; however, if present, the mortality rate is very high (up to 50%) [7]. The triad of renal failure, jaundice and splenomegaly was described as Weil disease in 1886 [8], and these symptoms were formerly considered to be the typical clinical picture of leptospirosis [3, 4]. Atypical manifestations and complications of leptospirosis are kidney or liver failure, myocarditis, pericarditis, pulmonary haemorrhage, gastrointestinal symptoms like pancreatitis, hematological and neurological symptoms [9, 10].

Direct microbiological diagnosis includes culture and molecular biological methods like polymerase chain reaction (PCR). In addition, serology has its role as an indirect approach using micro-agglutination testing (MAT), which is the gold standard; however, such tests are typically available only at specific centers and are not applied in many cases. Furthermore, the application of an ELISA assay is possible [1, 11]. For the treatment of leptospirosis, there are no consistent guidelines so far. In mild cases, oral doxycycline (2 × 100 mg/day per os for 7 days) is recommended. In severe cases, intravenous penicillin G (i.v. 1.5 million units/6 h for 7 days) or ceftriaxone (i.v. 1 g/day for 7 days) is the drug of choice [3].

## Case report

A 32-year-old otherwise healthy man was admitted to a ward for internal medicine in October 2016. He complained of fever (up to 39 °C), pain in the right leg as well as tingle in the right arm for just 15 min at home. There were no other pathological findings or neurological symptoms. During physical examination, a little redness at the right lower leg was found. Laboratory tests revealed increased inflammatory parameters [leukocytes 12.4 gpt/l, C-reactive protein (CrP) 141.3 mg/l, procalcitonin 43.07 ng/ml]. An empiric treatment with ceftriaxone (2 g/day) was initiated due to the fever of unknown origin. X-ray of the thorax showed no pathological findings. Due to the neurologic symptom, i.e., tingle in the right arm, a lumbar puncture was performed and revealed unremarkable results (total number of cells 1.00 Mpt/l; protein 248.60 mg/l; glucose 4.28 mmol/l). Duplex sonography showed no stenosis of the brain supplying vessels. During magnetic resonance imaging of the head, a 1 cm diffusion disorder in the splenicum of the corpus callosum was found, and in the context of infection without clear genesis, a transesophageal echocardiography was performed. A floating structure (11 × 7 mm) in the area of the ascending aorta and a broad base structure at the mouth of the vena cava superior (35 × 10 mm) were observed. Consequently, endocarditis with left-brain transient ischemic attack was suspected. On day 2 of hospitalization, an increase of the retention parameters [creatinine 159 µmol/l, glomerular filtration rate (GFR) 46.76 ml/min] as well as the appearance of diarrhea was recognized. The stool cultures showed no pathological findings. The retention parameters were improved during volume substitution.

**Fig. 1:**
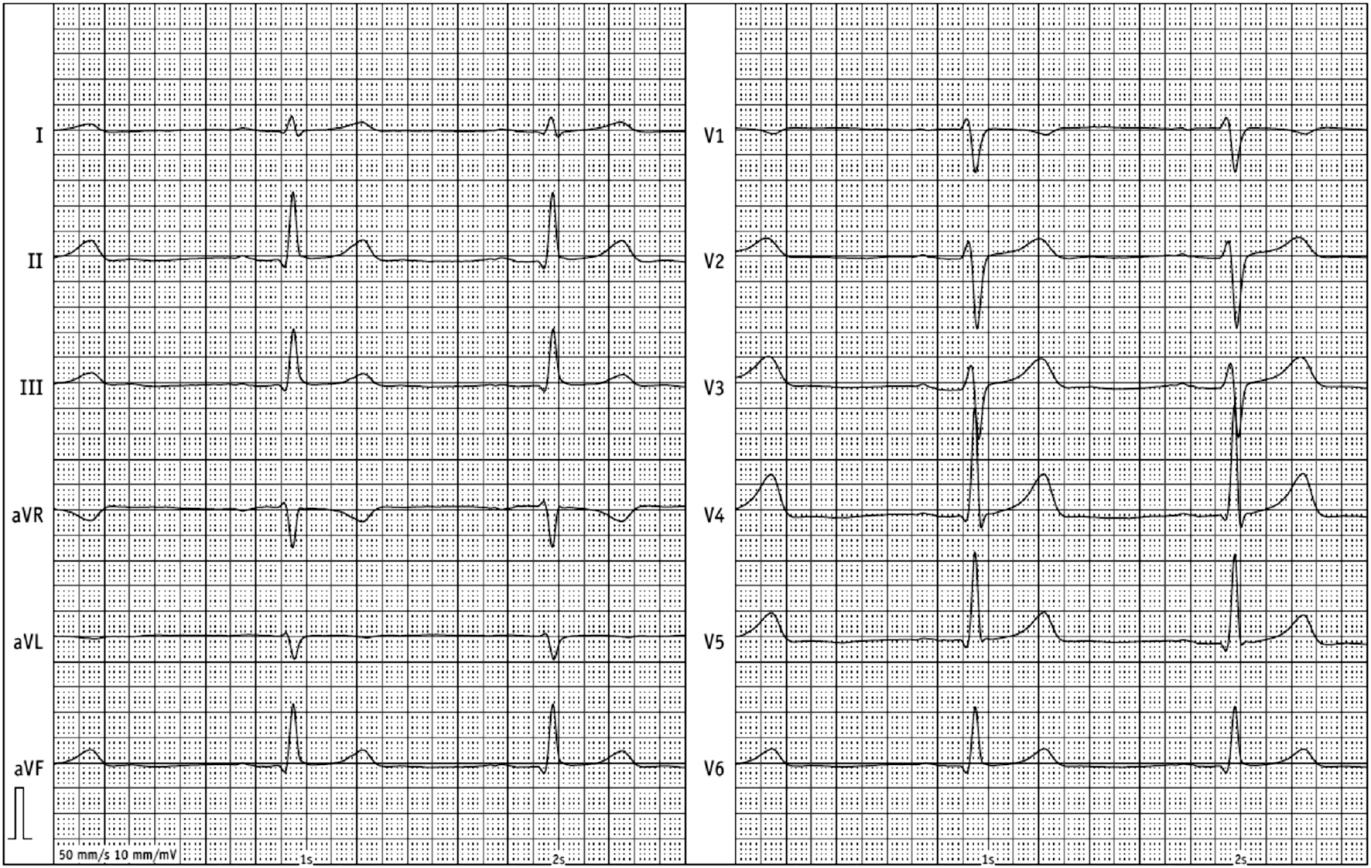
Electrocardiogram with saddle-back ST segment elevations in II, III, aVF, V_4_–V_6_

**Fig. 2:**
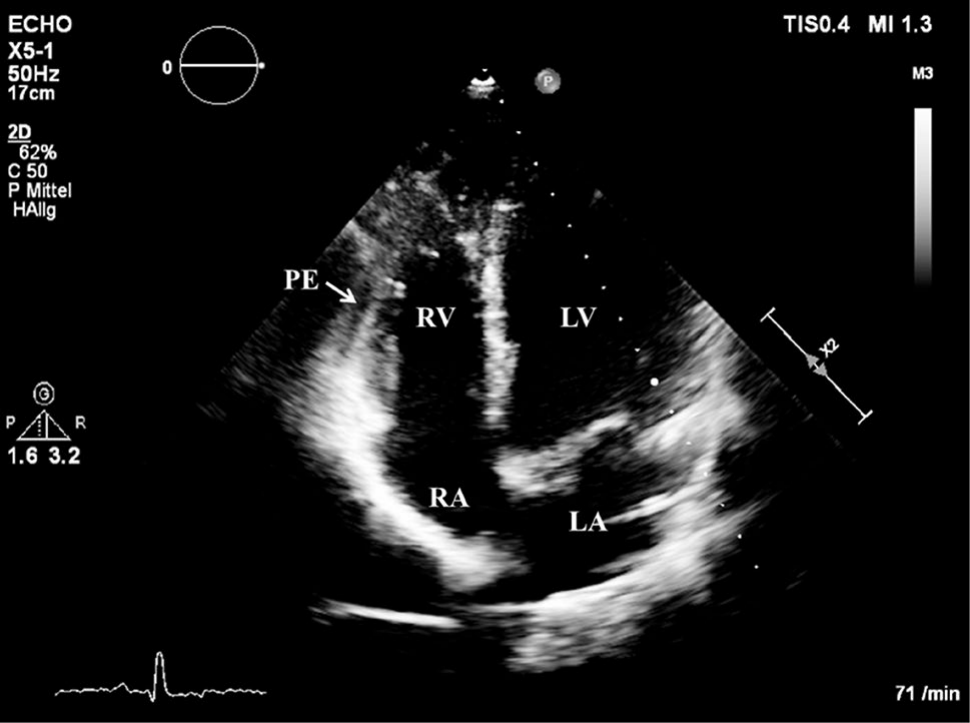
An echocardiography showed a pericardial effusion [PE]. Right ventricle [RV], left ventricle [LV], right atrium [RA], left atrium [LA]

For further treatment of a suspected endocarditis, the patient was transferred to the intermediate care unit at university hospital Jena on day 2 of hospitalization. After the transfer to the department of cardiology in Jena, an electrocardiogram was performed. Saddle-back-like ST segment elevations were observed in the leads II, III, aVF and V_4_–V_6_ (Fig. 1). A troponin I [TnI] correlate was not present and the patient did not feature any symptoms of a cardiac infarction; consequently, a coronary angiography was not performed. A repeated transesophageal echocardiography could not confirm the vegetations mentioned above; however, a pericardial effusion (end-diastolic 5–6 mm in front of the right ventricle) was found (Fig. 2). Computer tomography of the abdomen revealed no focus of infection and magnetic resonance imaging of the heart excluded a myocarditis but confirmed pericardial effusion. The B-type natriuretic peptide (BNP) was very mildly increased up to 115 pg/ml. In conclusion, a pericarditis had to be diagnosed. Due to suspected endocarditis and in the absence of pathogen detection, the initial antibiotic strategy with ceftriaxone was empirically changed to gentamicin (1 × 240 mg/day), ampicillin/sulbactam (4 × 3 g/day) and flucloxacillin (4 × 2 g/day) on day 1 and to gentamicin (320 mg/day) and ampicillin/sulbactam (4 × 3 g/day) on day 3. Based on antibiotic therapy, the general state of health improved and the inflammation parameters decreased (CrP 27 mg/l, procalcitonin 5.3 ng/ml) but fever persisted. The liver function parameters increased (aspartate aminotransferase [ASAT] 3.29 µmol/(l × s), alanine aminotransferase [ALAT] 6.97 µmol/(l × s)). A Holter electrocardiogram over 24 h was inconspicuous for relevant cardiac arrhythmias.

As no conclusive cause of infection could be found by extensive examination, consultation was performed by the infectious diseases consultation service on day 6 and a specific risk stratified anamnesis was performed. The patient reported having participated in a cross-country run with fresh water and mud contact 14 days before the onset of symptoms. Considering this anamnesis in the context of the present symptoms and the striking laboratory parameters, flu-like symptoms as well as kidney failure and pericarditis, the diagnosis of leptospirosis was proposed. The screening test of leptospirosis was positive (agglutination test: titer 1280, normal < 320) and Leptospira-specific immunoglobulin M as well as immunoglobulin G was detected (on day 8). Furthermore, the MAT in serum was positive for *Leptospira kirschneri* (titer 1:100). However, PCR on day 10 of hospitalization using urine and blood was negative.

Antibiotic therapy was switched to ceftriaxone (2 g/day for 7 days) with consecutive normalization of transaminases and body temperature. The infection parameters as well as the fever normalized during ceftriaxone therapy. The initial ST elevation was not present anymore. After 14 days in total, the patient was discharged from the hospital with improved general state of health. After 14 days from his discharge, the polymerase chain reaction in blood was negative for leptospirosis. In the echocardiography, no pathological findings could be revealed and the computed tomography of the heart was also inconspicuous one month after discharge. A second MAT screening as well as a testing of immunoglobulin M and G was negative 3.5 years later.

## Discussion

Leptospirosis is transmitted all over the world. Due to the diversity of symptoms—asymptomatic to lethal outcome—a high number of undetected cases is suspected [3, 4]. Similar to our case, it is often difficult to establish the diagnosis due to missing information within the anamnesis. Furthermore, the symptoms of leptospirosis can result in a completely different diagnosis such as dengue fever, hepatitis, influenza, hantavirus infection, yellow fever, typhoid fever and many more [3, 5, 8, 12]. In our case, we also observed symptoms like fever, increasing liver and retention parameters as well as diarrhea, which are also typically symptoms of other diseases. A detailed anamnesis and awareness of leptospirosis is, therefore, essential [3, 4].

High-risk groups for leptospirosis are sewer workers, field workers and veterinarians due to their contact with contaminated water and host animals. In Germany, leptospirosis is an occupational disease for these groups [3]. In Germany in 2006 and in Austria in 2010, outbreaks of leptospirosis at triathletes were described through the contact to contaminated water [13, 14]. The same infection route was found in our case. Our patient was probably infected during a cross-country running near Fürth (Bavaria, Germany).

Besides a detailed anamnesis and clinical examination, laboratory tests are the diagnostic cornerstone of leptospirosis [3]. Likewise, in our case, there was an increase of renal function parameters and liver function parameters. For leptospirosis, damage of the kidneys to acute renal failure is typical [3, 4]. The increase of liver values, especially of bilirubin, is also a manifestation of leptospirosis. After change to ceftriaxone, the liver function parameters decreased immediately. It remains unclear whether the decline in liver enzymes arose from the discontinuation of triple antibiotics or the treatment of the leptospirosis [3, 4].

As shown in our case report, the agglutination test [1, 11], was clearly positive with a titer of 1:1280 (normal < 320). Thus, a fourfold increased value was measured supporting the diagnosis leptospirosis according to the WHO [15] criteria as well as those of the Robert Koch Institute [RKI] [3]. Furthermore, the leptospirosis-specific IgM-ELISA was positive, which is an additional diagnostic criterion of the WHO and the RKI [3, 15]. The standard test for leptospirosis, the MAT, confirmed the finding and revealed a titer of 100 for *Leptospira kirschneri* in a serum sample taken on day 8 of hospitalization, which further supports the diagnosis according to the WHO criteria [15]. However, this sample was tested 3.5 years later after hospitalization. The agglutination test verifies the diagnosis of leptospirosis from the beginning of the second disease week, before that the result might be negative despite the presence of infection. Immunoglobulin M instead can be detected already during the first week of disease [1, 3, 11] and the MAT features a high sensitivity (82%) in the second week of illness [11]. An additional test (PCR) was performed after 10 days (in blood and urine) as well as after 4 weeks (in blood) after the onset of the symptoms, which both were negative. Sensitivity of the PCR using either blood or serum is very high (100%) within the first four days of illness. In the interval of 5–10 days after the first symptoms, the sensitivity decreases to values of 69% (to 100%) showing that the number of false-negative results increases with longer infection times [16]. Consequently, in our present case, the PCR was presumably performed too late; thus, a negative result was found.

The initial suspected diagnosis was an endocarditis with a left-brain transient ischemic attack. The first echocardiography revealed suspect findings interpreted as vegetations in the context of infective endocarditis. However, the echocardiographic diagnosis of endocarditis is very challenging due to the heterogeneity of additional structures or intra-cardiac masses even when using transesophageal echocardiography [17]. Consequently, the interpretation of echocardiographic findings in the context of suspected endocarditis, in particular in case of atypical extra-valvular manifestations, is highly challenging and requires a lot of experience. The subsequent echocardiography at the university hospital could not confirm this finding; however, a small hemodynamically irrelevant pericardial effusion without indication for pericardiocentesis was found. This is an interesting finding since changes in echocardiography are rarely documented in leptospirosis cases [18]. The patient was hospitalized with a high-fever infection from an external hospital to the intermediate care unit of the university hospital. Here, the electrocardiogram revealed saddle-back-like ST segment elevations in II, III, aVF, V_4_–V_6_. Considering all clinical, laboratory and imaging findings, these changes in the electrocardiogram had to be interpreted as a non-specific sign in terms of leptospirosis-associated pericarditis. Nevertheless, although the detected electrocardiographic pattern is considered typical for peri- and/or myocarditis, especially when occurring in the majority of leads in resting electrocardiogram, it is not very specific and a variety of differential diagnoses, i.e., acute coronary syndrome or broken heart syndrome, have to be taken into account [19, 20].

In general, cardiac involvements in leptospirosis are rare.[18, 21]. However, exact percentages have not been reported so far. Changes in the electrocardiogram occur especially as atrial fibrillation, atrioventricular block or nonspecific repolarization. In literature, there is a range from 48 to 70% for changes in electrocardiogram by leptospirosis [4, 9, 10, 21]. A myocarditis is even rarer than abnormality in echocardiographic or electrocardiogram. However, exact percentages are not provided [9, 10]. In our case, the patient had, in addition to the changes in the electrocardiogram, a pericardial effusion and an increased BNP. In summary of these findings, a pericarditis could be diagnosed. Pericarditis is also an atypical manifestation of acute leptospirosis. However, there are no specific reported numbers how often a pericarditis is associated with leptospirosis [9, 10]. After the therapy with ceftriaxone for one week, the pathological symptoms regressed within 1 month.

In conclusion, to be able to initiate the right therapy as early as possible, it is increasingly important to consider a diagnosis of leptospirosis for non-risk groups performing outdoor activities who present with flu-like symptoms and changes in electrocardiogram and/or pericardial effusion.
